# Validity of *Xiphophorus* fish as models for human disease

**DOI:** 10.1242/dmm.050382

**Published:** 2024-02-01

**Authors:** Manfred Schartl, Yuan Lu

**Affiliations:** ^1^ The Xiphophorus Genetic Stock Center, Department of Chemistry and Biochemistry, Texas State University, San Marcos, TX 78666, USA; ^2^ Developmental Biochemistry, Theodor-Boveri Institute, Biocenter, University of Würzburg, Würzburg 97074, Germany

**Keywords:** Cancer, Emerging models, Epistasis, Expression dysregulation, Hybrid incompatibility, Obesity

## Abstract

Platyfish and swordtails of the genus *Xiphophorus* provide a well-established model for melanoma research and have become well known for this feature. Recently, modelling approaches for other human diseases in *Xiphophorus* have been developed or are emerging. This Review provides a comprehensive summary of these models and discusses how findings from basic biological and molecular studies and their translation to medical research demonstrate that *Xiphophorus* models have face, construct and predictive validity for studying a broad array of human diseases. These models can thus improve our understanding of disease mechanisms to benefit patients.

## Teaser

**Summary:** We review how well-established and emerging *Xiphophorus* fishes and their interspecies hybrids can be used for studying human diseases, to highlight research opportunities enabled by these unique models.

## Introduction

When thinking about the features of a model organism, several criteria immediately come to mind. An animal model should be well suited for addressing the scientific questions of interest under laboratory conditions. Easy to breed, genetically defined strains should be available, and the husbandry should be economic. Sufficient research tools such as cell lines, antibodies and molecular probes should be available. Genomic resources, such as a high-quality reference genome sequence and appropriate databases for the most up-to-date omics approaches, are a must. Transgenesis and genome modification techniques should be established for analyses of gene and protein function. Finally, adoption of a research organism also requires an active scientific community that cooperates in establishing publicly accessible databases and in exchanging knowledge and materials. To make the research feasible at all, opportunities for funding should exist.

Although these criteria are the precondition, a model organism for biomedical research is determined by its validity. Three levels have to be fulfilled: (1) face validity (i.e. does the model replicate human clinical findings?), (2) construct validity (i.e. do the molecular, genetic, cellular and physiological mechanisms in the model reflect mechanisms of the human disease?) and (3) predictive validity (i.e. can the model predict currently unknown aspects of human disease?).

A diverse array of animal models that include both invertebrates and vertebrates has been established. Besides the widely known laboratory animals such as mice, rats and zebrafish, many more species have shown their value for studying certain aspects of a human disease or being of even wider applicability. This includes several fish species, and platyfishes and swordtails of the genus *Xiphohoru*s are among them.

*Xiphophorus* is a genus of central American freshwater fishes. To date, 26 species that live in various freshwater habitats in the Atlantic drainage of Mesoamerica, from Northern Mexico to Guatemala, have been described. All species are relatively small, ranging in size from 3.5 to 16 cm and thus are well suited for keeping in aquaria (Kallman and Kazianis, 2006). Ornamental breeds of three species, green swordtail (*Xiphophorus hellerii*), southern platyfish (*Xiphophorus maculatus*) and variable platyfish (*Xiphophorus variatus*), with spectacular coloration and fin shapes are well known to aquarists and fish hobbyists, and can be found in every pet shop. Their unique adaptive phenotypes and the possibility to experimentally produce interspecies hybrids make them an advantageous system to address questions in ecology, evolution, development, physiology and behavior (Earley, 2006; Powell et al., 2020, 2021; Rosenthal and De León, 2006). To render *Xiphophorus* a research organism, we have established highly inbred genetic lines, cell lines and a large collection of genomic resources (see Box 1). However, because of the livebearing mode of reproduction, which makes it difficult to obtain early-stage embryos and then raise them until term, transgenic technologies are not yet available.

**Box 1. The *Xiphophorus* Genetic Stock Center**

The *Xiphophorus* Genetic Stock Center (XGSC; https://www.xiphophorus.txst.edu) was established in 1939 at Cornell University. It was relocated to Texas State University in 1993. The center currently hosts 24 of 26 known *Xiphophorus* species that are categorized in 61 pedigreed lines and eight different interspecies hybrids, and has the capacity to produce 29 types of interspecies hybrids.

Pedigree lines are maintained in over 1400 aquaria. XGSC maintains stocks that represent both highly inbred lines (e.g. *X. maculatus* Jp163A is 119th generation inbred) and genetic variability across species. Furthermore, the XGSC has a repository for literature on *Xiphophorus* and provides access to genomic and transcriptomic resources.

**Table:**
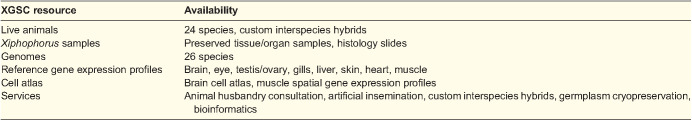


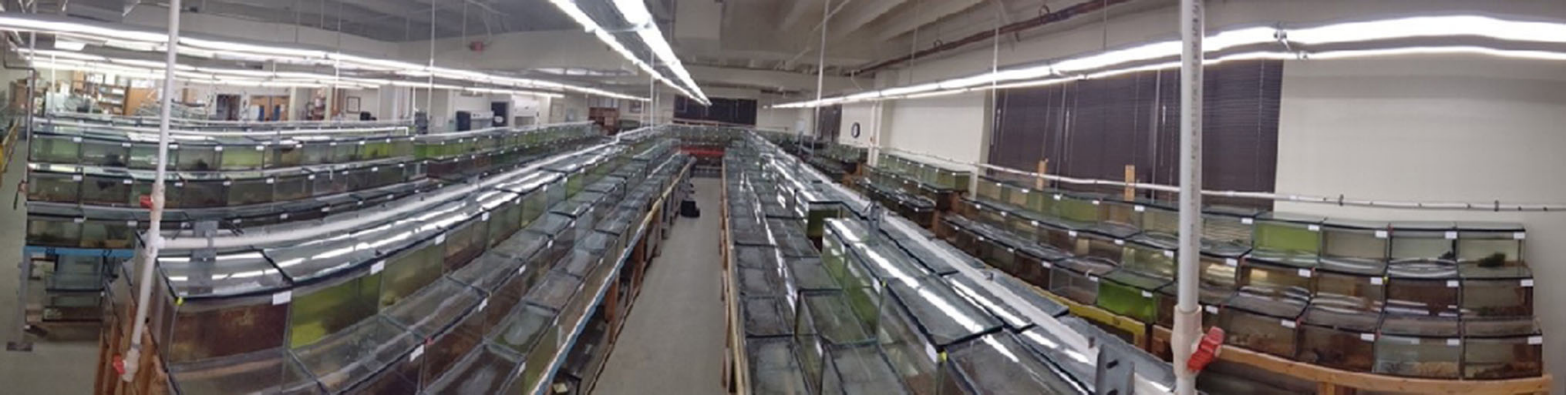

**Panoramic view of the XGSC fish room.**

In this Review, we present examples of how *Xiphophorus* fish species can be used as models for diverse human disease and discuss the main features and findings from these examples through the lens of face, construct and predictive validity.

## The *Xiphophorus* melanoma model

### The classical cross and other hybrid models

**Fig. 1.**
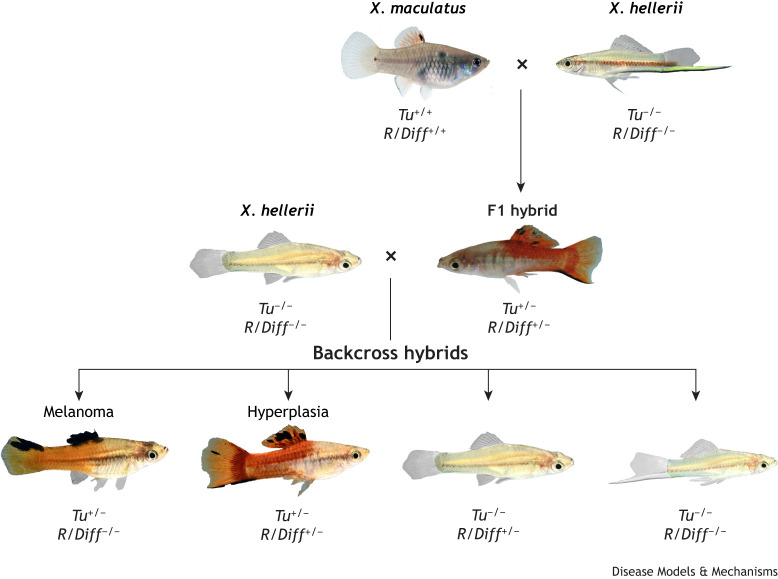
**The Gordon–Kosswig–Anders cross.** The crossing procedure results in hyperpigmentation and in the formation of melanoma in platyfish–swordtail hybrids. The substitution of platyfish *Xiphophorus maculatus* chromosomes containing the tumor-suppressing locus *R/Diff* with the *R/Diff*-free chromosomes of the swordtail *Xiphophorus hellerii* results in F1 hybrid fish with one copy of *R/Diff* and insufficient suppression of the tumor gene *Tu*. These animals show benign hyperpigmentation. Backcrossing the hyperpigmented F1 hybrids with *R/Diff*-free *X. hellerii* results in four possible hybrid genotypes. One quarter of these backcross segregants recapitulate the genotype of F1 hybrids and thus show benign hyperpigmentation, whereas backcross segregants that have inherited only *R/Diff*-free chromosomes develop highly malignant melanoma due to unobstructed activity of *Tu*. −/−, absence of gene; +/−, heterozygous condition; +/+ homozygous condition. Photo credit: Markita Savage, Lindsey Sanchec, Manfred Schartl and Yuan Lu of XGSC.

*Xiphophorus* is the one of the oldest animal systems for studying melanoma. Almost a century ago, it was discovered that certain platyfish and swordtail hybrids, which belong to different *Xiphophorus* species, develop highly malignant melanoma (Gordon, 1931; Häussler, 1928; Kosswig, 1928). Mendelian genetic analyses formalized the classical ‘Gordon–Kosswig–Anders’ cross (Anders, 1991) and thus established this model (Fig. 1). The establishment of this model is based on the observation that individuals of *X. maculatus* exhibit black pigmentation spots, e.g. in the dorsal fin. The spots are composed of a certain type of giant melanocyte, the macromelanophores, and show all features of human melanocytic nevi (Regneri et al., 2019), which consist of proliferative, often multinucleated melanocytes that are in contact with each other and form small cell aggregations. Usually, nevi in humans and fish alike are benign but can transform into skin cancer under certain conditions. In particular, heritable factors that manifest as multiple mole syndromes strongly increase the risk of benign nevi transforming into cutaneous melanoma in humans (Chang et al., 2009). Thus, they are regarded as premalignant pigment cell lesions.

The occurrence of nevus-like spots in the platyfish were explained by the action of a ‘*tumor*’ gene (*Tu*), which is typically suppressed by another gene, called ‘*regulator*’ (*R*) or ‘*differentiation*’ (*Diff*) (Schartl and Walter, 2016). We refer to it as *R/Diff* hereinafter. Due to this control, the malignant action of *Tu* is restricted and only permits the appearance of the nevus-like, benign pigment spots. Individuals of the *X. hellerii* that exhibited no macromelanophore spots were postulated to have no *Tu* and, consequently, to also be devoid of *R/Diff* (Anders et al., 1984). Because *Tu* and *R/Diff* are located on different chromosomes, they become segregated when platyfish are crossed to swordtails (Schartl and Walter, 2016). In the classical Gordon–Kosswig–Anders cross, a *X. maculatus*/*X. hellerii* F1 fish is backcrossed to *X. hellerii*. The offspring from this backcross segregate according to Mendelian laws into four groups (Fig. 1) (Anders et al., 1984; Walter and Kazianis, 2001). The fish that have inherited only *Tu* but not *R/Diff* develop malignant melanoma. These tumors grow exophytically and are highly invasive, eventually leading to death.

Besides this classical cross, several other hybrid melanoma models were developed over the years, involving *Xiphophorus andersi* and *Xiphophorus couchianus* as *Tu-* and *R/Diff-*free partners for the crossing-conditioned elimination of *R/Diff* (Walter and Kazianis, 2001). Instead of using the Jp163A spotted-dorsal (Sd) strain, an inbred platyfish line with spots on the dorsal fin, three of these models use the Jp163B spotted-side (Sp) strain, a sibling inbred line exhibiting macromelanophore spots on the body side, as the *Tu*- and *R/Diff*-carrying parent. Furthermore, backcross hybrids with *X. hellerii* as the recurrent parent, similar to the classical Gordon–Kosswig–Anders cross, are of special note because they develop the benign premalignant condition melanosis in the first *Tu*-carrying backcross generation even in the absence of *R/Diff*. Melanosis in these backcross hybrids covers the entire body sides. This can progress to malignant melanoma only after ultraviolet B (UVB) irradiation of adult fishes and of juvenile ones soon after birth (Nairn et al., 2001; Setlow et al., 1989; Walter and Kazianis, 2001). UVB irradiation was also shown to induce cyclobutene pyrimidine dimers at a higher frequency than the 6–4 photoproduct in *Xiphophorus signum* (Walter et al., 2014). Both types of damage to the DNA could be repaired photoenzymatically in the presence of visible light or via nucleotide excision repair in the absence of light (Mitchell et al., 2001). However, the nucleotide excision repair pathway was less efficient in interspecies F1 hybrids (Mitchell et al., 2004).

The generally accepted genetic hypothesis for explaining melanoma formation in *Xiphophorus* hybrids is that the crossing-conditioned elimination of a tumor-suppressing allele, *R/Diff*, contributes to the macromelanophore pattern in the *Tu*-expressing parental lineage. However, as discussed in Garcia-Olazabal et al. (2023 preprint) and in a previous publication from our group (Schartl, 1995), it should be noted that the resulting phenotypes can similarly be explained by attributing the melanoma-inducing *Tu* activity in the hybrid genome to the presence of not yet characterized intensifying genes contributed by chromosomes from the parent that does not have the black spots. To our knowledge, no crossing experiment so far has confirmed either of these two hypotheses.

The occurrence of melanoma in *Xiphophorus* is not restricted to hybrids. Although rarely observed, melanoma can develop in purebred species (Fernandez and Morris, 2008; Kazianis and Borowsky, 1995; Schartl et al., 1995). Those tumors develop from the nevus-like spots and are almost exclusively seen in mature males of advanced age. As testosterone treatment experiments in hybrids have previously shown a melanoma-promoting effect of the androgenic steroid (Schartl et al., 1982; Siciliano et al., 1971), it is conceivable that these spontaneous melanomas in purebred fish are associated with the higher androgen levels of dominant males.

Several attempts have been made to use *Xiphophorus* hybrids as models for cancer development after exposure to carcinogens (Anders et al., 1991; Kazianis et al., 2001a,b; Schwab et al., 1978, 1979). These studies used known carcinogens with a strong mutagenic action and indeed resulted in the induction of a broad spectrum of different cancer histotypes, which recapitulated many of the known human tumor entities. Some genotype–tumor type correlations were noted, indicating the face validity of *Xiphophorus* as a cancer model, but these studies were not taken further and await to be revisited with current molecular analytical tools and deep-sequencing genomics methods. Some strains of *Xiphophorus* developed other types of neoplasia, e.g. ocular and thyroid tumors, even in the absence of carcinogens (Gorbman and Gordon, 1951; Gordon, 1946; MacIntyre and Baker-Cohen, 1961), but these findings were not pursued further.

### Xmrk and signal transduction in the melanoma cell

The melanoma-inducing *Tu* locus was mapped to the sex chromosomes of the platyfish. It was determined to be tightly linked to the sex determination locus and to other sex-linked traits (Anders, 1991; Kallman, 1975b). Using a reverse genetic approach employing restriction fragment length polymorphisms between *Tu*-containing, *Tu*-mutant and wild-type fish, Wittbrodt et al. (1989) isolated a candidate gene thought to be the critical component of the *Tu* locus. It encodes a mutant duplicated version of the epidermal growth factor receptor. To distinguish it from the proto-oncogene *egfrb*, it was designated *xmrk* for *Xiphophorus* melanoma receptor kinase. Functional evidence from studies in which *xmrk* was expressed under the promoter of the pigment cell-specific *mitfa* gene of medaka (Schartl et al., 2010) supports the notion that *xmrk* is indeed a tumor gene. The transgenic fish developed malignant pigment cell tumors with 100% penetrance, showing that *xmrk* is sufficient for melanoma development. The proof that *xmrk* is also necessary for tumor formation came from mutant hybrid fish that had lost the capacity for melanoma formation. In these mutants, the *xmrk* gene was disrupted by a transposon insertion and, consequently, no functional gene product was expressed (Schartl et al., 1999).

The *xmrk* gene is a local duplicate of *egfrb*, one of the two Egf receptor orthologs (ohnologs) of teleost fish. In the process of gene duplication, *xmrk* acquired a novel upstream promoter region (Adam et al., 1993; Regneri et al., 2015) and several amino acid changes. Two changes in the extracellular domain of the receptor are responsible for its dimerization via an intermolecular cysteine bridge, leading to this variant receptor being ligand independent in its signaling capacity (Dimitrijevic et al., 1998; Gomez et al., 2001, 2002; Meierjohann et al., 2006). Hence, Xmrk is a constitutively active receptor that continuously transmits intracellular signals to confer an autonomous proliferative state and other hallmarks of malignancy to the *Xiphophorus* melanocytes (Meierjohann and Schartl, 2006; Schartl and Walter, 2016).

**Fig. 2.**
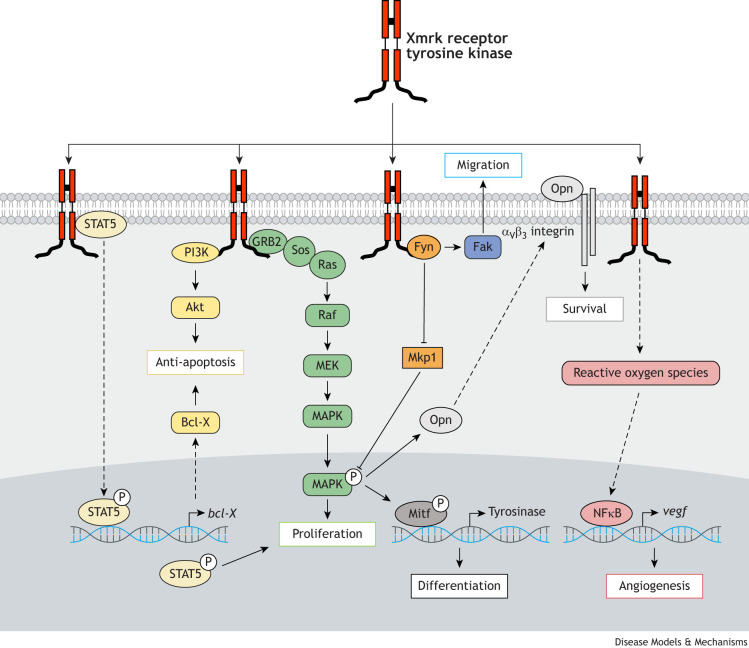
**Signal transduction pathways downstream of the Xmrk receptor tyrosine kinase.** The *xmrk* gene is the driving oncogene that initiates melanoma in *Xiphophorus*. Signaling pathways downstream of Xmrk relay the constitutive activity of the membrane-bound receptor into gene expression programs that drive several components of the neoplastic phenotype. Xmrk promotes proliferation, differentiation, angiogenesis, migration and anti-apoptosis signaling pathways.

The most important downstream signaling pathway activated by the Xmrk receptor tyrosine kinase is the Ras/Raf/MAPK pathway (Meierjohann and Schartl, 2006) (Fig. 2), also known as the mitogenic cascade because it mediates proliferation of the cell. Xmrk also activates the STAT5 pathway, which feeds into proliferation as well (Hassel et al., 2008). Avoidance of apoptosis is important for cancer cell survival and is also managed by Xmrk through its activation of phosphatidylinositol 3-kinase (PI3K)-Akt signaling, aided by STAT5-enhanced expression of Bcl-X (Morcinek et al., 2002). Through MAPK, Xmrk activates the transcription factor Mitf (Delfgaauw et al., 2003; Wellbrock et al., 2002), which is the main regulator of melanocyte differentiation and proliferation. Usually, melanocytes cannot survive when translocated to the deeper layers of the skin because of a death signal elicited by extracellular matrix components of the dermis (Li and Herlyn, 2000; Montgomery et al., 1994). Xmrk induces the secretion of osteopontin (Opn), which blocks the receptor for these pro-death extracellular matrix fragments and allows the melanoma cells to invade the dermis (Geissinger et al., 2002). Melanoma cell migration itself is controlled through the kinase Fak, which is activated by the Xmrk-associated cytoplasmic kinase Fyn (Teutschbein et al., 2009; Wellbrock et al., 2002). Finally, the formation of new blood vessels to support tumor growth is initiated by NFκB and reactive oxygen species (ROS) (Schaafhausen et al., 2013), which are a byproduct of the Xmrk-induced neoplastic phenotype of the melanoma cells.

Another feature of the multitasking oncogenic ability of Xmrk is that a differential quantitative output of the signal has different effects. Weaker signals keep melanocytes in the proliferative state, whereas strong signaling drives them into senescence, which is mediated by pronounced oxidative stress (Leikam et al., 2008). Activation of oncogene-induced senescence also produces multinucleated giant cells, a long-known histological feature of nevi.

Being strategically placed upstream of many intracellular signaling pathways that are involved in manifesting the neoplastic phenotype, *xmrk* is a highly potent driver oncogene. This is best demonstrated by transgenic experiments in zebrafish, which has no *xmrk* ortholog, wherein the expression of *xmrk* cDNA was controlled by a liver-specific promoter. The transgenic zebrafish developed rapidly growing, highly malignant hepatocarcinoma with 100% penetrance (Li et al., 2012).

The capacity of transgenically expressed *xmrk* to promote cancer development in non-melanocytic cells and the findings that *xmrk* expression is seen not only in macromelanophore spots, but also in gills, eyes and brain (Schartl et al., 1999), pose the question as to why *xmrk* only induces pigment cell tumors in *Xiphophorus*. This could be due to cell type-specific growth properties or differences in *xmrk* expression control. Transcriptional regulation studies of the 200 kb genomic region flanking *xmrk* provided no answer to this question (Regneri et al., 2015), leaving further characterization of the *xmrk* promoter or distant regulators open for future research.

### The quest for *R/Diff*

The oncogene *xmrk* was identified more than 30 years ago and its biochemical function in transforming a normal melanocyte into a cancer cell are reasonably well understood. This is contrasted by *R/Diff*. Locating the *R/Diff* locus, using molecular marker linkage studies of benign melanocytic lesions in *Tu* (*xmrk*)-carrying hybrids, to chromosome 5 (Morizot and Siciliano, 1983) led to the proposition of a first candidate gene, *cdkn2ab* (or *cdkn2x*) (Kazianis et al., 1999). This cyclin-dependent kinase inhibitor gene was mapped to the same chromosomal region as the *R/Diff* effect, and its human ortholog (*CDKN2A*) is a well-known tumor suppressor (Jiao et al., 2018) that is frequently affected, particularly in melanoma. *CDKN2A* is mutated or mis-expressed in about 10% of all melanoma cases (http://cancer.sanger.ac.uk/cosmic), with germline mutations occurring in up to 40% of familial melanoma cases (Rossi et al., 2019), and appears to be a primary mediator of senescence in human nevi (Ross et al., 2011). Accordingly, overexpression of *Xiphophorus cdkn2ab* in *xmrk*-transformed melanoma cells induced senescence, leading to reduced proliferation (Regneri et al., 2019). Coexpression of *Xiphophorus cdkn2ab* in medaka transgenic for the *mitfa:xmrk* melanoma-inducing gene resulted in full suppression of melanoma development, whereas CRISPR/Cas9-mediated knockout of the endogenous *cdkn2ab* in the *mitfa:xmrk* transgenic medaka strongly enhanced tumor growth (Regneri et al., 2019). This provided the functional proof that *cdkn2ab* can act as a potent tumor-suppressing gene in fish melanoma. In line with this are findings from *in vitro* studies that established a link between Xmrk signaling strength and senescence induction (Leikam et al., 2008). However, these results do not conclusively show that *cdkn2ab* is indeed the critical gene encoded by the *R/Diff* locus. Other than for *Tu*, no loss-of-function *Xiphophorus* mutants have been isolated, which would be informative for showing whether a gene candidate is necessary for the development of benign lesions instead of malignant melanoma.

Two swordtail species, *Xiphophorus malinche* and *Xiphophorus birchmanni*, form natural hybrids in several habitats in east-central Mexico (Culumber et al., 2011). In some populations, these hybrids develop melanoma early in life driven by the *xmrk* oncogene (Powell et al., 2020, 2021). This cancer-initiating gene is derived from the parental species *X. birchmanni*, which exhibits a macromelanophore pattern in the tail fin. Like in the artificial laboratory hybrids discussed above, the pigment lesions of the natural hybrids can be classified as either benign or malignant, indicating the action of a *R/Diff* analog. Genome-wide association and admixture mapping approaches identified *adgre5*, also named *cd97*, as a candidate regulator of the *R/Diff* benign pigmentation phenotype. The ortholog of *adgre5* in mammals plays a role in epithelial metastasis and is associated with tumor invasiveness (Safaee et al., 2013; Ward et al., 2018). This second candidate gene for *R/Diff* also maps to a distinct region on chromosome 5, about 7 Mb from the region identified in the Gordon–Kosswig–Anders cross towards the telomeric region. A recent preprint describes *in vitro* experiments monitoring the proliferation and migration of mouse melanocytes expressing either the *X. malinche* or the *X. birchmanni adgre5*, revealing that the *X. birchmanni* gene clearly suppressed growth and migration to a higher extent than the *X. malinche* version (Garcia-Olazabal et al., 2023 preprint). In the same preprint, embryos from the *mitf:xmrk* transgenic melanoma medaka line that were injected at the one-cell stage with the *X. birchmanni adgre5* transgene driven by a pigment cell-specific promoter displayed only a few transformed pigment cells, whereas those expressing the *X. malinche adgre5* did not show this reduced transformation effect (Garcia-Olazabal et al., 2023 preprint). Of note, these data indicate that the *X. birchmanni* allele has a tumor-suppressing activity compared to the *X. malinche* allele. This can be taken to indicate that *R/Diff* – at least in the natural *X. birchmanni*/*X. malinche* hybrids – conforms to the genetic hypothesis that melanoma forms due to crossing-conditioned elimination of a locus that keeps the *xmrk* oncogene in check.

In parallel to the *X. malinche*/*X. birchmanni* natural hybrids study, separate work using a combination of genome sequencing and association analyses in the backcross hybrids from the classical Gordon–Kosswig–Anders cross fine-mapped *R/Diff* to a ∼100 kb interval on linkage group 5 containing three genes (Lu et al., 2020). This region is 6 Mb away from *cdkn2ab* towards the chromosome 5 centromere and 13 Mb away from *adgre5*. Expression analyses using RNA sequencing showed that only *rab3d* is expressed in the nevus-like spots of the dorsal fin and in the benign and malignant melanocytic lesions of the backcross hybrids (Lu et al., 2020). Like for *cdkn2ab* and *adgre5*, the final validation of *rab3d* as the causal gene underlying the *R/Diff* locus effect requires transgenic or genome-editing experiments in *Xiphophorus*. These approaches are currently thwarted by the lack of established techniques in these viviparous fish. The existing mapping and expression analyses, however, establish *rab3d* as another strong candidate gene for the *R/Diff*-mediated phenotype.

Besides the dominantly acting oncogenes and the recessive tumor suppressor genes that initiate and drive the neoplastic state, the so-called tumor modifier genes are determinants of the course of the disease, even though they are not primarily involved in the emergence of a tumor. *R/Diff* falls under this definition. In the heterozygous state, it prevents the progression of melanocytic lesion to the malignant state. The identification of different *R/Diff* candidate genes depending on the genetic background in which *xmrk* is expressed puts the *Xiphophorus* system forward for the study of melanoma modifiers, which would give the model predictive validity. Even in heavily scrutinized mouse models, tumor modifier genes have been notoriously difficult to identify. This is because modifiers are often pleiotropic, have individual-specific effects, provide only partial, albeit critical, contributions to the disease phenotype and can easily escape detection in cancer genome-sequencing projects.

## The *P*-locus and regulation of the onset of the reproductive period

In several species of *Xiphophorus*, males exhibit a clear phenotypic polymorphism in the timing of puberty, which marks the onset of the reproductive phase. As males cease to grow when they become reproductively mature, this is linked to differences in body weight and size. This polymorphism is associated with a number of other traits, including reproductive strategies, dominance and territorial behavior. Natural populations exhibit a wide spectrum of male phenotypes, ranging from early-maturing, very small males that adopt sneak-mating behavior, to late-maturing large males, which are territorial and preferred by females due to their large body size and elaborate courtship behavior. Genetic studies revealed that the entire suite of male phenotypes is determined by alleles at a single locus (Kallman, 1989; Kallman and Borkoski, 1978) on both the X and Y sex chromosomes. This locus was called the ‘puberty’ or ‘pituitary’ (*P*) locus because it was reasoned that the gene acts in the pituitary gland as the main regulator of puberty and reproductive physiology (Schreibman and Kallman, 1977). In each population, several alleles exist that determine the age of puberty onset. Thus, besides the environmental conditions that influence growth and nutritive status, a strong genetic component regulates the beginning of the male reproductive phase in *Xiphophorus*.

Molecular studies revealed that both sequence and copy number variation of the *mc4r* gene underlies the *P*-locus polymorphism (Lampert et al., 2010). The *Xiphophorus mc4r* genes encode two G protein-coupled melanocortin receptor isoforms. The A-type receptors are wild type and act biochemically in the same way as other known vertebrate melanocortin receptors, including those of humans (Lampert et al., 2010; Liu et al., 2020, 2019). They are expressed in the hypothalamus and bind to pituitary-derived hormones, the melanocortins. After melanocortin binding, A-type Mc4r elicits a cyclic AMP response in the cell, which signals to the genome. The mammalian MC4R is critically involved in regulating appetite and the metabolic response to food uptake (Kühnen et al., 2019). Mutations in murine and human *MC4R* result in severe obesity (Farooqi et al., 2003; Itoh et al., 2011). In the Mexican cavefish *Astyanax mexicanus*, mutations in *mc4r* convey adaptation to the nutrient-poor cave environment (Aspiras et al., 2015). The reduced basal activity and reduced maximal response of these mutated receptors elevate fish appetite, growth and starvation resistance.

The A-type alleles of *Xiphophorus* are present in both males and females and are located on the X chromosome (Lampert et al., 2010). In addition, a second isoform type of *mc4r* is encoded by the B-type alleles found only on Y chromosomes. Due to various degenerative mutations, these B-type receptors are deficient in either ligand binding or signal transduction (Lampert et al., 2010). Large males have high copy numbers of B-type alleles and high *mc4r* expression. The B-type isoforms are co-expressed with A-type isoforms (Liu et al., 2020) and decrease intracellular cAMP signaling after ligand exposure (Lampert et al., 2010). A- and B-type receptors were shown to form heterodimers in the cell membrane (Liu et al., 2023), which has led to a molecular explanation for how the Mc4r system regulates puberty, wherein the B-type isoforms exert a dominant-negative effect on A-type isoforms (Lampert et al., 2010; Liu et al., 2023). This lowers the signaling output of the melanocortin system in the hypothalamus. It was hypothesized that a certain threshold of this signal must be reached during the juvenile period to initiate puberty, and the B-type isoform-mediated lowering of the signal promotes the initiation of puberty. The findings in *Xiphophorus* thus link energy metabolism and growth regulated by Mc4r in the hypothalamus to sexual maturation regulated by the hypothalamus–pituitary–gonadal axis.

The molecular mechanisms and biochemical features of the *mc4r* system in *Xiphophorus* reflect what is known from mammals and thus provide another argument for its construct and predictive validity as a human disease model.

## Epistatic dysregulation in *Xiphophorus* hybrids uncovers human disease genes

### Post-zygotic incompatibility and epistatic dysregulation

The genetic changes during speciation have been a topic of intense research in evolutionary biology for almost two centuries. Pre-zygotic and post-zygotic isolation mechanisms shield gene flow between diverged species. Hybrid progeny of divergent populations, especially interspecies hybrids that occur accidentally in nature or artificially in the laboratory by overcoming pre- and/or post-zygotic isolation are of the utmost importance for evaluating the impact of epistasis among alleles belonging to each population. The hybrids can, in some cases, exhibit advantageous phenotypes over either parental population, i.e. heterosis or hybrid vigor, whereas in other cases, they suffer from hybrid dysfunction or negative heterosis (East, 1936; Hou and Schacherer, 2016; Khan et al., 2011; Semel et al., 2006; Sohail et al., 2017; Soyk et al., 2017). The Dobzhansky–Muller–Bateson model states that when diverged alleles are recombined in interspecies hybrids, they can create negative epistasis, which is the underlying cause of hybrid dysfunction (Orr, 1996), an overall drawback of interspecific hybrid progeny due to the emergence of unfit traits. It has been recognized that both a failure in regulating gene expression properly (i.e. dysregulation) and faulty gene expression (i.e. misregulation) contribute to out-of-range transcriptional traits and are significantly involved in hybrid dysfunctions. Epistasis among divergent cis and trans gene regulatory elements such as promoters, enhancers and transcription factor-binding sites can lead to gene expression dysregulation, which gives rise to hybrid dysfunction (Civetta, 2016; Mack and Nachman, 2017; Ortiz-Barrientos et al., 2007). Therefore, gene expression dysregulation, used synonymously for both dysregulation and misregulation in this Review, connects genetic incompatibility to hybrid dysfunction.

Aberrant molecular genetic alteration is a precursor of disease. The transcriptional phenotype, which is defined as the collection of disease-associated gene expression patterns, can be used as a diagnostic tool even in model animals that do not yet exhibit visible disease phenotypes (Klotz et al., 2019, 2018; Lu et al., 2019). Disease is clearly an unfit trait that contributes to the overall hybrid dysfunction phenotype. Therefore, interspecies hybrids can be used to screen for human disease-relevant molecular phenotypes. In addition, characterizing regulators of disease genes enables the detection and early medical intervention through comparative genomics.

### Signaling pathways affected by epistatic dysregulation

One of the unique features of *Xiphophorus*, not shared by other established animal models, is the almost unlimited opportunity to produce not only inter-strain and inter-populational hybrids, but also hybrids and backcross hybrids of different species, even from the most phylogenetically distant branches of the genus.

Molecular phenotypes can be studied in two types of interspecies hybrids: the F1 interspecies hybrid, produced by natural breeding or artificial insemination of females of one species with male gametes of a different species, and the backcross interspecies hybrid, resulting from breeding the F1 interspecies hybrid to one of the parental species. The interspecies hybrids that were intensively studied for epistatic dysregulation are limited to *X. maculatus*/*X. hellerii*, *X. maculatus*/*X. couchianus* and *X. couchianus*/*X. hellerii* hybrids. Thus, in this section, we will only focus on the findings from these three crosses (Lu et al., 2018b, 2015, 2017, 2023).

Several studies performed on the Amazon molly (*Poecilia formosa*), *Drosophila* hybrids and *Xiphophorus* interspecies hybrids (Dedukh et al., 2022; Lu et al., 2021, 2015) revealed that, with respect to the global gene expression landscape, both parental alleles contributed more or less equally to the expression of the hybrid genome. This was documented for both naturally occurring hybrids and for hybrids produced in the laboratory by enforced breeding. However, at the locus-specific level, hybrid gene expression can exhibit one of the following scenarios: the expression level matches that of one parental species; the gene is expressed at an intermediate level of both parental species; or the expression is transgressive, i.e. either lower or higher than in either parental species. Only transgressive expression results from cis and trans regulatory element divergence (Lu et al., 2023) and is thought to be the result of novel interactions between divergent trans and cis regulatory elements (Landry et al., 2002; Mack and Nachman, 2017; Sanchez-Ramirez et al., 2021).

In a study of the aforementioned *X. maculatus*/*X. hellerii*, *X. maculatus*/*X. couchianus* and *X. couchianus*/*X. hellerii* hybrids, whole-body transcriptome profiling comparisons of each parental species to their interspecies F1 hybrids showed that various genes exhibited transgressive expression in hybrids (Lu et al., 2023). A portion of the dysregulation could be traced to interspecific single-nucleotide polymorphisms and short insertions/deletions within the upstream regulatory sequences, structural variants such as long insertions/deletions affecting the coding and regulatory regions, and transposable elements affecting transcriptional start sites or upstream regulatory regions. However, a detailed mechanistic understanding of how this regulatory region variance affects gene expression in the hybrids is lacking.

**Fig. 3.**
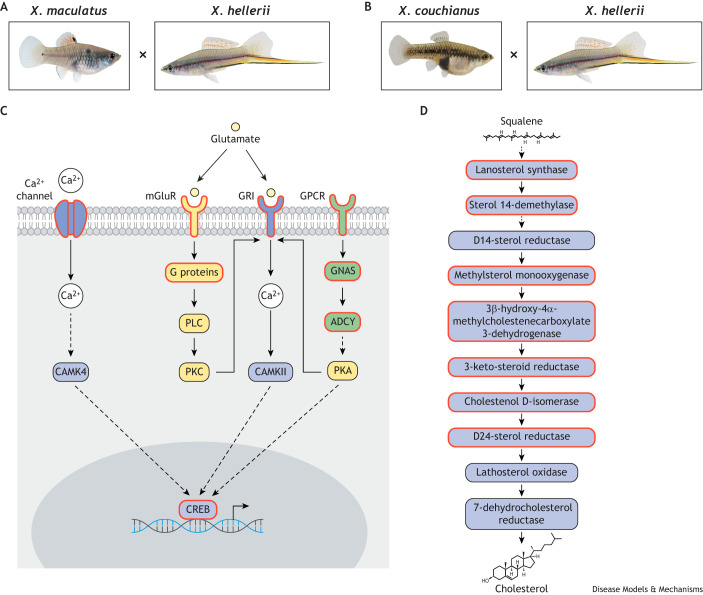
**Examples of signaling pathways showing dysregulated expression patterns in *Xiphophorous* hybrids.** (A,B) *X. maculatus*/*X*. *hellerii* (A) and *X. couchianus*/*X. hellerii* (B) F1 hybrids (Lu et al., 2023). (C) Neuronal pathway. (D) Cholesterol pathway. The dysregulated genes are highlighted by red outlines.

Individual hybrids exhibited distinct, hybrid-specific signaling pathway deregulation (Fig. 3), but the hybrids also shared some common dysregulated pathways. Dysregulated genes in *X. couchianus*/*X. hellerii* hybrids are mainly involved in cholesterol metabolism (Fig. 3B,D). In *X. maculatus*/*X. hellerii* hybrids, in addition to the cholesterol biosynthesis pathways, the highly dysregulated pathways are related to neuronal function (Fig. 3A,C). Lastly, dysregulated pathways in *X. couchianus*/*X. maculatus* hybrids were highly similar to those identified in *X. maculatus*/*X. hellerii* hybrids, but without a high representation of neuronal pathway dysregulation (Lu et al., 2023). For a full list of all dysregulated pathways and genes in these three hybrids, see Table S1. Based on these findings, the interspecies hybrid can be implemented as a discovery tool for gene dysregulation.

It is possible to disentangle the genetic network of cis and trans transcriptional regulators underlying gene dysregulation using the quantitative trait locus (QTL) method. This usually relies on creating a population of advanced backcross hybrids, determining genetic variant site genotypes, profiling their gene expression, forming a linear model between the genotype of each variant site and the expression of each gene, followed by association analysis using analysis of variance (ANOVA) and genome-wide false discovery rate (FDR) correction. A QTL study performed in a *X. maculatus*/*X. hellerii* backcross hybrid population, the *X. hellerii*×(*X. maculatus*/*X. hellerii*) backcross, identified skin QTLs associated with expression variation of more than 300 genes (Lu et al., 2018b). Most of these were under cis regulation. This is consistent with findings from other model organisms and humans (Lu et al., 2018a).

### Disease type and human disease genes uncovered from *Xiphophorus* hybrids

As demonstrated by the aforementioned spontaneous melanoma development in *X. maculatus*/*X. hellerii* hybrids (Fig. 1), disease can be an unfit trait contributing to the overall hybrid dysfunction. The F1 hybrids are considered disease ‘predisposed’, a condition in which genomes exhibit global heterozygosity. Disease-relevant morphological and molecular phenotypes can arise owing to changes in the copy number of modulating alleles that, when fully functional, suppress disease. Therefore, such hybrids allow researchers to screen for phenotypical changes that are associated with human disease. It is expected that in advanced hybrids, e.g. the backcross, intercross or outcross hybrids, loss of heterozygosity of a disease-modulating gene will allow full penetrance of a disease phenotype (for genotypic combinations, see Table S2), as exemplified by the *X. maculatus*/*X. hellerii* hybrids in which the F1 hybrids (*xmrk^+/null^; Diff^+/−^*) exhibit pigment cell hyperplasia, whereas the subsequent loss of *Diff* in select backcross hybrids (*xmrk^+/null^; Diff^−/−^*) leads to melanoma.

A recent study compared the dysregulated genes of the *X. maculatus*/*X. hellerii*, *X. maculatus*/*X. couchianus* and *X. couchianus*/*X. hellerii* F1 interspecies hybrids to human disease-relevant genes (Lu et al., 2023). Among these, an average of 6% of genes dysregulated in the hybrids were related to human diseases. Because this study investigated whole-animal transcriptomics rather than organ- and tissue-specific profiles, it identified disease-related genes that affect several organs. For example, *CLDN4* is overexpressed in human ovarian cancer (Litkouhi et al., 2007) and its *Xiphophorus* ortholog is dysregulated in *X. maculatus*/*X. hellerii* hybrids. Furthermore, aberrant high expression of *CASR* is observed in inflammation, vascular calcification, atherosclerosis, myocardial infarction, hypertension and obesity (Sundararaman and van der Vorst, 2021), and the *Xiphophorus* ortholog of *CASR* is overexpressed in the *X. maculatus*/*X. hellerii* hybrid. Finally, *GCK* inactivation causes maturity-onset diabetes of the young (Gloyn, 2003), and its *Xiphophorus* ortholog exhibits the same suppression in *X. couchianus*/*X. hellerii* hybrid. These findings indicate that the outcomes of genetic incompatibility in the *Xiphophorus* hybrids are relevant to human disease and underscore the construct validity of *Xiphophorus* as a human disease model.

## Emerging *Xiphophorus* models

Here, we describe examples of emerging disease models in *Xiphophorus* fish that are in the early stages of development and have not been fully assessed for their face, construct and predictive validity.

### Albinism

**Fig. 4.**
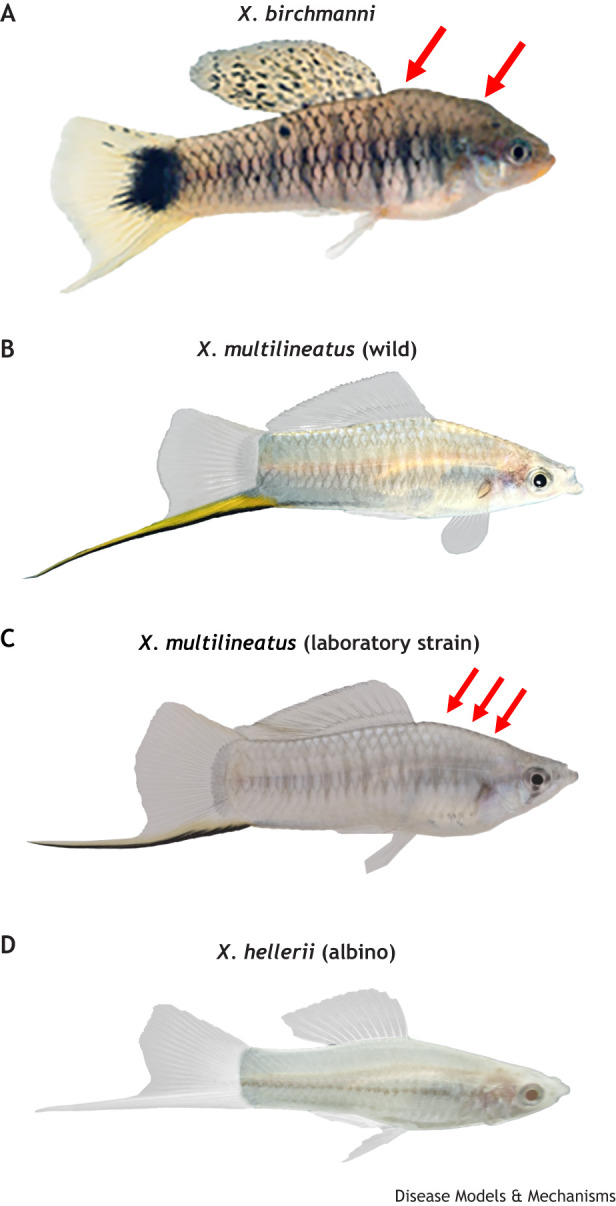
**Emerging models in *Xiphophorus*.** (A) Adult male of *X. birchmanni* with nuchal hump (arrows). (B) *X. multilineatus* male from a wild population without hump. (C) A male from an overfed laboratory strain of *X. multilineatus* with nuchal hump (arrows). (D) A male from an albino mutant *X. hellerii* strain. Photo credits: *Xiphophorus* Genetic Stock Center (XGSC) (Box 1).

Wild *X. hellerii* has vibrant body colorations that are characterized by green- or orange-colored sword tails in the male. Albinism is a rare condition in *Xiphophorus* fish, where individuals lose the black and dark brown eumelanin-associated coloration traits, including black stripes on the sword (the male ornament), micromelanophore spots between fin rays, and melanin granules in the retinal pigment epithelium. A select strain of *X. hellerii* exhibits albinism that is characterized by no pigmentation in the eyes and skin (Fig. 4D). The *X. hellerii* albinism is similar to human oculocutaneous albinism type 2 (OCA2), and inactivation of the *OCA2* gene product is known to cause albinism in humans (Kamaraj and Purohit, 2014). The molecular mechanism of how *X. hellerii* develops albinism is currently being studied.

### Micromelanophore pigmentation pattern

Multiple platyfish species develop several caudal fin pigmentation patterns (Borowsky, 1984; Culumber, 2014; Kallman, 1975a). By studying an intercross hybrid population produced by crossing two species in which a crescent-shaped caudal fin pigmentation pattern is either present (*X. maculatus*) or absent (*X. couchianus*), we identified a region on chromosome 17 that is associated with the caudal fin pigmentation pattern, which is hypothesized to be involved in neural crest differentiation and migration (Culumber, 2014).

### Fin regeneration

The caudal fin of many fish species has been recognized as particularly suitable for regeneration studies because it is easily accessible for surgery. Imaging and morphometric analyses can also be conducted without technical caveats (Akimenko et al., 2003). Anatomical investigations of various teleost fish species showed that the caudal fin develops from tissue that is below the notochord column and that it is a ventral appendage, despite its external dorsal–ventral symmetry (Desvignes et al., 2018; Hadzhiev et al., 2007; Sallan, 2016). However, *X. maculatus* has been shown to diverge from this typical caudal fin arrangement (Rees et al., 2022) in that its caudal fin includes principal rays derived from the mesenchyme above the notochord column, rendering the *Xiphophorus* an innovative model system to study skeletal development and regeneration (Rees et al., 2023). The most important observation from *Xiphophorus* caudal fin regeneration following amputation is that the regenerating fin can faithfully reproduce the original bifurcation points, unlike the zebrafish, which cannot reproduce the ray-branching pattern following amputation (Akimenko et al., 2003; Azevedo et al., 2011, 2012). The regeneration of wounded tissue following caudal fin amputation in *Xiphophorus* is driven by BMP signaling. Fin regeneration relies on the formation of lepidotrichia and actinotrichia, with the latter being the primary dermal skeleton structure to support blastemal budding. BMP signaling can restore *Xiphophorus* lepidotrichia and actinotrichia, consistent with observations in zebrafish (Rees et al., 2023). Rees et al. (2023) also used *X. maculatus* to investigate the role of Tp63, a pivotal transcription factor in mammalian epidermis development, differentiation and regeneration. They found that Tp63 is expressed in the quiescent basal layer of the wounded epidermis in the regenerating *Xiphophorus* fin. Tp63 is known to maintain the quiescence of basal epidermal cells (Su et al., 2009). Therefore, this work suggests that the function of Tp63 in *Xiphophorus* is consistent with that in mammals, whereas its role in zebrafish fin regeneration is not known.

### An evolutionary model for diet-induced obesity

Humans have evolved to cope with alternating abundance and scarcity of food (Dyson et al., 2006). Obesity is a risk factor for metabolic syndrome due to a maladaptation to constantly available or overabundant food sources.

Although fish do not develop an obesity phenotype, a protrusion in the nuchal region called nuchal hump is found in several species. The humps are diverse in structure and may exhibit different adaptive functions, e.g. predation avoidance, intraspecific sex recognition and species recognition (Barlow and Siri, 1997; Portz and Tyus, 2004; Takahashi, 2018). However, the nuchal humps serve as fat deposition sites in a few species, for example, *Cyphotilapia gibberosa*, *Cyphotilapia citrinellum* and *Cyrtocara moorii.* Adipogenesis was enhanced in nuchal hump tissue in *Cyrtocara moorii*, an African cichlid (Lecaudey et al., 2019; Tompkins et al., 2021).

In *Xiphophorus*, *Xiphophorus multilineatus* and *X. birchmanni* males exhibit nuchal humps (Fig. 4A,B). However, *X. multilineatus* do not naturally develop this phenotype. They only develop a nuchal hump in laboratory conditions after being fed a high-calorie diet (Fig. 4C), which suggests it is a result of maladaptation to a highly caloric diet, similar to the human condition. Unlike in wild *X. multilineatus*, the *X. birchmanni* nuchal hump may be an adaptation to changes in food availability in its natural habitat, i.e. the hump may arise during the warmest season when water temperature correlates to high food abundance (Culumber et al., 2012; Rauchenberger and Morizot, 1990).

*X. multilineatus* have shown different growth rates in response to diet, with males from an intermediate body size class, named Y-II in literature, growing the fastest (Kallman, 1989; Zimmerer and Kallman, 1989; Morris et al., 2012). Accordingly, the size of the *X. multilineatus* nuchal hump is influenced by both genotype and diet: males from the Y-II size class develop the largest nuchal humps relative to their body size, and males from the slower-growing Y-s size class develop the smallest nuchal humps. Additionally, individuals with large nuchal humps exhibit higher body mass index (BMI). Histological examination of the nuchal hump showed that the tissue consists of lipid-filled adipocytes, recapitulating typical adipose histology (Tompkins et al., 2021).

Comparative transcriptomic analyses of high- and low-calorie-diet-fed Y-II *X. multilineatus* males showed differential expression of genes relevant to appetite control, metabolism, diabetes, energy and lipid regulation (Lu et al., 2017), supporting the notion that the nuchal hump is a maladaptive phenotype to a highly caloric diet and provides a valuable model for human obesity. Future studies should focus on delineating the relationship between nuchal hump size, genetics, BMI and metabolism to further validate *X. multilineatus* as a model for human obesity.

## Validation of findings from *Xiphophorus* as a model for human diseases

### Construct validity

**Fig. 5.**
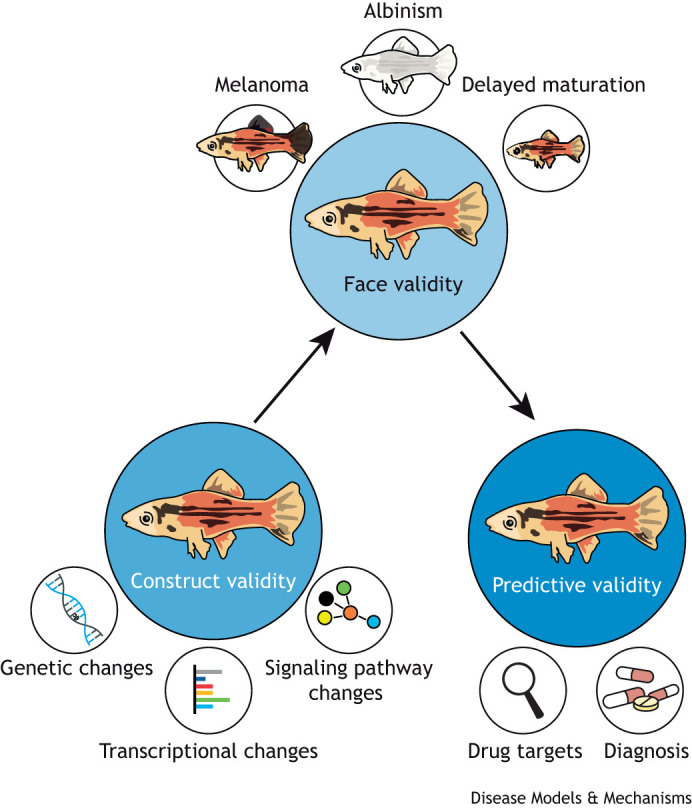
Different aspects of validity of the *Xiphophorus* models for human diseases.

As we discussed in the Introduction to this Review, the first level of validation is construct validity, which tests whether the molecular, genetic, cellular and physiological mechanisms in the model reflect mechanisms of the human disease (Fig. 5). The pathohistology of *Xiphophorus* tumors is very similar to that of human malignant melanoma (Gimenez-Conti et al., 2001; Riehl et al., 1984), and these tumors grow and metastasize after subcutaneous transplantation to nude mice (Schartl and Peter, 1988). Their gene expression profiles also largely overlap with those of advanced human melanoma (Lu et al., 2018a). As we discussed above, the *Xiphophorus* melanoma driver oncogene *xmrk* is an *EGFR* homolog. *EGFR* is mutated in 10% of human melanoma and is an important initiator of cancer development in up to 50% of malignant glioma, lung cancer and many other cancers (Uribe et al., 2021). Xmrk activates the same biochemical pathway that was found to be critical for the autonomous proliferation of tumor cells in up to 70% of human melanoma (Yang et al., 2023).

With respect to the tumor suppressor mechanism, the human homologs of all three *R/Diff* candidate genes, *cdkn2ab*, *rab3d* and *adgre5*, are recurrently affected in human cancer. The homologues of *cdkn2ab* are potent tumor suppressor genes and are mutated or deleted in a high percentage of human melanoma and many other cancers (Jiao et al., 2018; Rossi et al., 2019). The *rab3d* and *adgre5* homologs have been associated with the malignant phenotype of several human cancers (Mehner et al., 2014; Safaee et al., 2013; Ward et al., 2018), reflecting that *Xiphophorus* tumors recapitulate pathways that are relevant to malignant progression in human cancer and thus have high construct validity for modeling human cancer.

Development of the *Xiphophoru*s models for the regulation of puberty onset and the related research on dietary-induced obesity are only at early stages. However, they have already shown construct validity: mutations in *Mc4r* cause metabolic dysregulation and obesity in the major mammalian disease model, the mouse, and in a large cohort of human patients (Farooqi and O'Rahilly, 2004; Itoh et al., 2011).

Although the characterization of the molecular mechanism is pending, studies of albinism in *X. hellerii* yielded the same gene underlying the human oculocutaneous albinism, i.e. *Xiphophorus oca2* and human *OCA2*, providing another example of the construct validity of this model. Oca2 is a membrane transporter of tyrosine for melanin synthesis, and the *oca2* variant-driven albinism in *X. hellerii* could depend on either *oca2* mutation and/or aberrant expression (Grønskov et al., 2007; Kamaraj and Purohit, 2014).

A particular example of construct validity can be assigned to the even more recently established hybrid gene dysregulation models. The first few pilot studies have already revealed epistatic gene interactions in *Xiphophorus* hybrids that parallel human conditions from ovarian cancer and inflammation to vascular calcifications, atherosclerosis, myocardial infarction, hypertension and obesity (Lu et al., 2023).

### Face validity

The next level of model validation is its face validity (Fig. 5). The question here is whether the model does replicate the clinical findings of a human disease. Human cutaneous melanomas originate from epidermal pigment cells. In *Xiphophorus*, like in humans, skin melanocytes reside in the epidermis. This is different from the mouse and other commonly used mammalian models, in which the melanocytes reside in the hair bulb. In addition to recapitulating the epidermal anatomical origin of human melanoma, the progression stages of fish melanoma parallel human primary melanomas. They start from a nevus-like black pigmentation spot followed by a radial growth phase, which then progresses to the vertical growth phase during which the melanoma cells invade into the deeper layers of the skin.

Solar UV radiation during childhood is the most common etiology of human melanoma. UV-induced nodular melanoma in *Xiphophorus* therefore provides a valid model for this category of skin cancers. An obvious but barely understood clinical finding in human patients with melanoma is that sex and hormonal status influence the frequency and course of the disease (Bhari et al., 2022). In *Xiphophorus*, the sex-linked higher malignancy of pigment lesions in males and the treatment experiments of melanoma-carrying fish with sex steroids replicate this phenomenon. The observed correlation between hormone status and melanoma development motivates further research on the hormonal status of patients. Moreover, better understanding of the relationship between puberty onset and melanoma development may improve our current understanding of melanoma pathophysiology (Caroppo et al., 2021).

Sexual maturation is another example of the face validity of the *Xiphophorus* model. Like in humans, nutritional status and onset of puberty are tightly linked in *Xiphophorus*. It has been shown that elevated BMI and obesity can delay puberty in boys (Soliman et al., 2014), and further research in *Xiphophorus* can help explain this link.

Notably, other emerging *Xiphophorus* models also recapitulate human disease phenotypes or symptoms. The *X. hellerii* albinism is consistent with human oculocutaneous albinism, i.e. no pigmentation in the eyes, skin and hair.

### Predictive validity

The third and most valuable aspect, from the translational point of view, is the predictive validity of a model (Fig. 5). An outstanding example of the predictive validity of *Xiphophorus* is that studies of oncogenic Xmrk signaling predicted the role of the receptor tyrosine kinase/Ras/Raf/MAPK pathway as the critical driver for melanoma a decade before this was confirmed in humans (Schartl and Walter, 2016). Today, it is well known that mutations in *BRAF* or *NRAS* are the drivers of up to 70% of human cancers, and therapies that target this pathway have been developed (Yang et al., 2023). Moreover, the secreted glycoprotein osteopontin was first identified in *Xiphophorus* melanoma as a key factor for the transition from radial to vertical growth (Geissinger et al., 2002). Osteopontin has since been included in diagnostic panels as a prognostic marker in clinical medicine (Kluger et al., 2011; Zhou et al., 2005). *Xiphophorus* studies also predicted a mechanism of interferon resistance in first-line melanoma treatments (Hassel et al., 2008; Wellbrock et al., 2005), and the finding that high-strength Xmrk signaling leads to an increase in ROS initiated studies that revealed a critical role for the enzyme cystothionase for melanoma growth. Pharmacological inhibition of this enzyme in human melanoma cells reconstituted senescence *in vitro* and knocking down expression of the gene that encodes cystothionase reduced tumor burden in a nude mouse transplantation model (Leikam et al., 2014).

The results from studies on the puberty gene predict a dominant-negative effect for heterozygous human carriers of an *mc4r* mutation. Studies on the *Xiphophorus P*-locus showed that the simultaneous presence of a mutant and wild-type Mc4r lowers the signaling output of the heterodimeric complex (Lampert et al., 2010; Liu et al., 2023). This finding predicts that humans who are heterozygous for one of the obesity-predisposing mutant *MC4R* alleles will also be at risk and may develop symptoms – albeit at a milder level than homozygous carriers of two mutant alleles – because heterodimers of the mutant form will interfere with the physiological function of MC4R.

## Conclusions

The research on cancer and reproductive maturation that we discussed in this Review substantiates the value of *Xiphophorus* as a model for human disease throughout all three phases of validation and continues to provide important scientific insight. New observations emerging from many gross and molecular phenotypes in *Xiphophorus* species and interspecies hybrids further show that the *Xiphophorus* system can be extended to model additional human diseases and health conditions.

## Supplementary Material

10.1242/dmm.050382_sup1 Supplementary information

Table S1. Disregulated genes and pathways in *Xiphophorus* hybrids.

Table S2. Expected genotypes for disease genes mapping.
