# Supplementary material for: Validity of Xiphophorus fish as models for human disease
Source: Dis Model Mech. 2024 Feb 1;17(1):dmm050382. doi: 10.1242/dmm.050382 (PMC10855230; doi:10.1242/dmm.050382)
Supplement: Supplementary information [file dmm-17-050382-s1.pdf]

Table S1. Disregulated genes and pathways in *Xiphophorus* hybrids.

Available for download at
https://journals.biologists.com/dmm/article-lookup/doi/10.1242/dmm.050382#supplementary-data

Table S2. Expected genotypes for disease genes mapping.

Available for download at
https://journals.biologists.com/dmm/article-lookup/doi/10.1242/dmm.050382#supplementary-data

Disease Models & Mechanisms • Supplementary information
